# Compensation of Physicians

**Published:** 1838

**Authors:** 


### Art. VIII.—Compensation of Physicians.

We return our thanks to those who have favored us, with statements of the rates of charging by themselves and the brethren in their immediate neighborhood; but the number of *data* is not yet great enough for generalization; we must, therefore, respectfully, request our patrons generally, at as early a day as may be convenient, to favor us with the information, asked for in our last number. It is our intention to make the whole the subject of an article in our July number. D.

